# A Reconfigurable Memristor-Based Computing-in-Memory Circuit for Content-Addressable Memory in Sensor Systems

**DOI:** 10.3390/s25206464

**Published:** 2025-10-19

**Authors:** Hao Hu, Yian Liu, Shuang Liu, Junjie Wang, Siyu Xiao, Shiqin Yan, Ruicheng Pan, Yang Wang, Xingyu Liao, Tianhao Mao, Yutong Chen, Xiangzhan Wang, Yang Liu

**Affiliations:** 1 State Key Laboratory of Electronic Thin Films and Integrated Devices, University of Electronic Science and Technology of China, Chengdu 610054, China; hu_hao@std.uestc.edu.cn (H.H.); liuyian@uestc.edu.cn (Y.L.); liushuang@uestc.edu.cn (S.L.); wangjunjie@uestc.edu.cn (J.W.); xiaosiyu@alu.uestc.edu.cn (S.X.); 202411311403@std.uestc.edu.cn (S.Y.); 202411311401@std.uestc.edu.cn (R.P.); 202412311214@std.uestc.edu.cn (Y.W.); 202411311405@std.uestc.edu.cn (X.L.); 202511311119@std.uestc.edu.cn (T.M.); 202511311118@std.uestc.edu.cn (Y.C.); wxz@uestc.edu.cn (X.W.); 2 Chongqing Institute of Microelectronics Industry Technology, UESTC, Chongqing 401332, China

**Keywords:** memristor, computing-in-memory, multi-bit processing, content-addressable memory, approximate matching

## Abstract

To meet the demand for energy-efficient and high-performance computing in resource-limited sensor edge applications, this paper presents a reconfigurable memristor-based computing-in-memory circuit for Content-Addressable Memory (CAM). The scheme exploits the analog multi-level resistance characteristics of memristors to enable parallel multi-bit processing, overcoming the constraints of traditional binary computing and significantly improving storage density and computational efficiency. Furthermore, by employing dynamic adjustment of the mapping between input signals and reference voltages, the circuit supports dynamic switching between exact and approximate CAM modes, substantially enhancing functional flexibility. Experimental results demonstrate that the 32 × 36 memristor array based on a TiN/TiOx/HfO_2_/TiN structure exhibits eight stable and distinguishable resistance states with excellent retention characteristics. In large-scale array simulations, the minimum voltage separation between state-representing waveforms exceeds 6.5 mV, ensuring reliable discrimination by the readout circuit. This work provides an efficient and scalable hardware solution for intelligent edge computing in next-generation sensor networks, particularly suitable for real-time biometric recognition, distributed sensor data fusion, and lightweight artificial intelligence inference, effectively reducing system dependence on cloud communication and overall power consumption.

## 1. Introduction

In recent years, with the rapid development of the Internet of Things (IoT) and edge computing, there has been an increasingly urgent demand for energy-efficient and low-latency computing in resource-constrained sensor nodes [1,2,3,4]. The traditional Von Neumann Architecture, which separates memory and computing units, requires frequent data movement during large-scale data-intensive tasks, resulting in high energy consumption and significant processing delays, which severely limits the performance of real-time edge computing [5,6]. Thus, Computing-In-Memory (CIM) architecture has emerged as a promising solution. By integrating computing units within or near memory, this architecture significantly reduces data access overhead and offers a new paradigm for energy-efficient edge computing [7,8,9,10].

Among various emerging memory technologies, memristors, also known as Resistive Random-Access Memory (RRAM), have garnered considerable attention in CIM circuits due to their small area, low power consumption, and compatibility with standard fabrication processes [11,12,13,14,15,16]. However, in most existing studies, memristors are primarily utilized as binary switching devices, with only two states representing “0” and “1”, thus failing to exploit their full potential for high-density information storage and processing [17,18,19,20,21]. In practice, certain types of memristors exhibit rich multi-level characteristics, being capable of achieving multiple stable resistance states [22,23,24]. Leveraging this feature in CIM circuits has the potential to substantially improve storage density and computational efficiency, making them particularly suitable for high-throughput edge applications such as biometric recognition and multi-sensor data fusion [25,26,27,28,29]. However, realizing such multi-bit CIM architectures requires overcoming challenges in both device control and circuit design [30,31,32].

This paper introduces a reconfigurable CIM circuit using RRAM to implement multi-bit Content-Addressable Memory (CAM) and Approximate Content-Addressable Memory (ACAM). Our proposed architecture achieves a higher storage density than its Complementary Metal-Oxide-Semiconductor (CMOS) counterparts and supports ACAM functionality through reconfiguring reference voltages. In the era of Large Language Models (LLMs), where data is commonly represented as embedding vectors, high-performance vector lookup and similarity searches are essential. A key challenge is to accelerate the process of finding the optimal vector within massive datasets on power-constrained edge devices. The proposed CIM-based ACAM targets this application, providing an efficient hardware solution.

The main contributions of this work include:

1. Exploitation of the multi-level behavior of memristors to enable multi-bit CAM, significantly enhancing storage density and computational parallelism;
2. A novel memristor read/write circuit design capable of achieving accurate and robust programming of memristor resistance states;
3. By flexibly configuring the reference voltages, the architecture enables reconfigurable CAM functionality, including CAM and ACAM.

The architecture of the paper is organized as follows: Section 2 presents the proposed RRAM CIM circuit architecture, read/write circuit design and the RRAM device. Section 3 systematically presents the electrical characteristics of the multi-bit memristor and waveform simulation results. Section 4 presents a discussion covering ACAM implementation analysis, performance evaluation, scalability challenges, and future perspectives. Finally, Section 5 summarizes the main contributions and research value of this work.

## 2. Methods

### 2.1. Architecture

**Figure 1:**
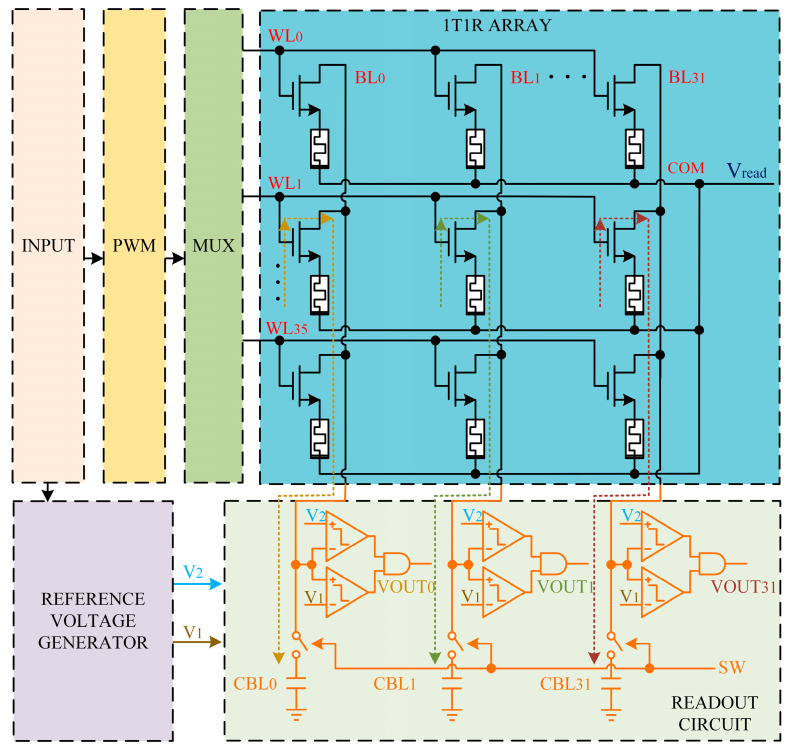
Schematic illustration of the proposed RRAM CIM circuit architecture.

Figure 1 shows the proposed RRAM CIM circuit for multi-bit CAM. The proposed circuit consists of the Pulse-Width Modulation (PWM) module, the Multiplexer (MUX) module, the One-Transistor-One-RRAM (1T1R) Array, the Reference Voltage Generator, and the Readout Circuit. The operational workflow is as follows: the input signal is fed to the PWM module to generate a pulse-width signal, which then drives the MUX to select a word line. At this point, the RC circuit formed by the memristor and the capacitor in the readout circuit begins charging. Simultaneously, the reference voltage generator provides the reference voltage required by the readout circuit.

1. The PWM module converts the 3-bit input signal into a pulse signal, whose duty cycle is proportional to the value of the 3-bit input. The modulated PWM signal is then fed into the MUX;
2. Based on the output from the PWM module, the MUX drives the corresponding word line high, selecting a specific row of memory cells;
3. The 1T1R array adopts a common-top-electrode structure composed of 1T1R unit cells. The word lines are set high for row selection by enabling the transistors, while the bit lines are used to access the memristors in a given column and are connected to the readout circuit;
4. The reference voltage generator produces two reference voltages based on the 3-bit input signal. These reference voltages are then used for subsequent comparison;
5. Using capacitors and voltage comparators, the readout circuit compares the bit-line voltage to reference voltages and yield the result.

### 2.2. Reference Voltage Generator

If the memristor exhibits at least eight stable and distinguishable conductance states, these states can be used to represent the 3-bit signals “000”, “001”, …, “111”. The capacitors connected to each bit line are designed with identical capacitance values, ensuring that the charging voltage accurately characterizes the different resistance states of the memristors, while applying distinct reference voltages at the voltage comparator stage enables differentiated output results, thereby achieving state discrimination.

**Figure 2:**
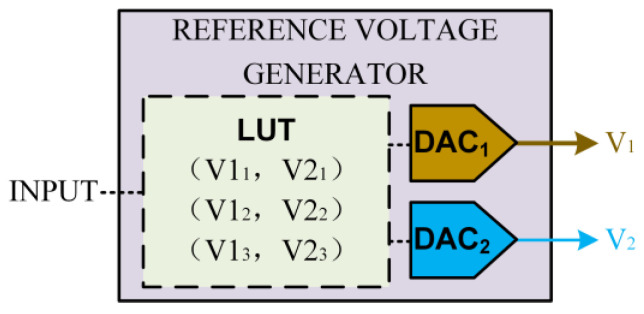
Schematic illustration of the Reference Voltage Generator.

The designed reference voltage generator in this circuit is illustrated in Figure 2.

In this structure, a Look-Up Table (LUT) is used to map the input signal to the corresponding reference voltages, which are then generated by two Digital-to-Analog Converters (DACs) and delivered to the readout circuit. In other words, the circuit output encodes the computation result of the input signal and the memristor′s conductance. By configuring the LUT, multi-bit CAM or ACAM can be implemented. Thus, the proposed circuit enables reconfigurable multi-bit CAM.

### 2.3. Memristor Read/Write Circuit

Conventional characterization of memristors typically requires the use of semiconductor parameter analyzers such as the Keithley 4200-SCS, which can precisely output voltage and simultaneously measure current. These instruments are well-suited for evaluating the electrical characteristics of memristors operating as binary switching devices. However, they exhibit significant limitations when applied to the multi-level memristors studied in this work. Even with a complete parameterized characterization of the device, achieving precise configuration of multi-level states using such equipment remains highly challenging. Moreover, the use of bulky instruments is not conducive to flexible read/write operations in application scenarios such as integrated sensing systems. To address these issues, we have developed a portable memristor read/write circuit board.

**Figure 3:**
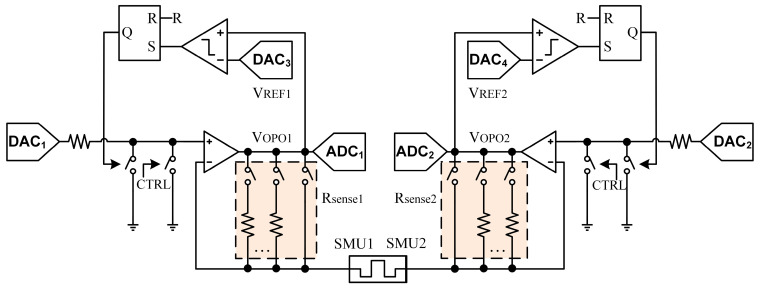
Schematic illustration of the Memristor Read/Write Circuit.

The proposed memristor read/write circuit in this work is illustrated in Figure 3. The circuit features a symmetric structure, with two symmetric sub-circuits connected to both terminals of the Device Under Test (DUT)—referred to herein, by analogy with the Keithley 4200-SCS, as SMU1 and SMU2. Without loss of generality, it can be assumed that SMU1 is connected to the top electrode of the memristor, while SMU2 is connected to the bottom electrode.

When performing a set operation on the memristor, a positive voltage must be applied to SMU1 while SMU2 is grounded. This is implemented as follows: *R_sense1_* is connected to a resistor with a magnitude comparable to that of the memristor (or, if the resistance is unknown, initially set to the minimum value), while *R_sense2_* is shorted. DAC_1_ outputs the set voltage value, and DAC_2_ outputs 0 V. The “virtual short” characteristic of the operational amplifier enables the set voltage to be applied directly across the DUT. The output voltage *V_OPO_* of the operational amplifier on the SMU1 side and the set voltage *V_set_* applied to the memristor satisfy the following relation:

*V_OPO_* = *I*·*R_sense1_* + *V_set_* (1)

where *I* denotes the current flowing through the memristor.

Based on this relationship, the current flowing through the memristor can be limited by setting an appropriate *V_REF1_*. When the current exceeds the preset limit, the value of *V_OPO_* will surpass *V_REF1_*, causing the output of the RS flip-flop to go high. This, in turn, pulls the potential at the SMU1 node to ground, thereby protecting the devices.

The minimum achievable pulse width of the read/write circuit is fundamentally limited by the finite voltage switching speed of the DAC. To mitigate this issue, a switch is added between the non-inverting input of the operational amplifier and ground, controlled by the CTRL signal. This allows the voltage at the SMU1 node to be pulled to ground immediately after the DAC completes its output, eliminating the delay associated with the DAC resetting its output.

When a reset operation needs to be performed on the memristor, a positive voltage must be applied to SMU2 while SMU1 is grounded. Due to the symmetric design of the circuit, the analysis is analogous to the set operation.

For a read operation, the output voltage *V_OPO_* of the operational amplifier on the SMU1 side and the read voltage *V_read_* applied across the memristor satisfy the following relation:

*V_OPO_* = *I*·*R_sense1_* + *V_read_* (2)

Since *V_OPO_* can be sampled by Analog-to-Digital Converter (ADC_1_), the current *I* can be derived from the above equation, enabling the calculation of the memristor’s resistance and thus realizing the read operation.

**Figure 4:**
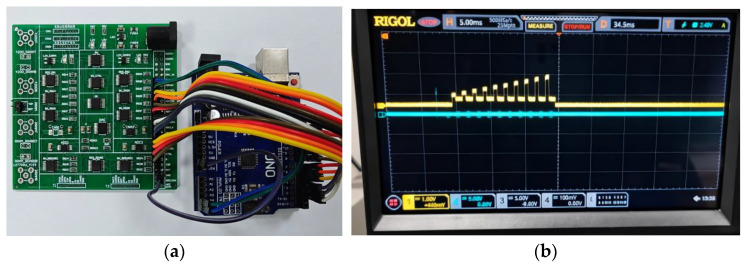
(**a**) Photograph of the read/write circuit prototype; (**b**) Voltage waveform of the memristor SET scan.

The physical implementation of the circuit is shown in Figure 4a, wherein a Microcontroller Unit (MCU) is employed to manage read and write operations. Experimental results demonstrate that the proposed read/write circuit successfully performs both resistance programming and reading functions. The voltage waveform diagram obtained from scanning the SET process of the memristor is illustrated in Figure 4b.

Based on the above analysis, the core of the read/write circuit lies in the operational amplifier. The LMV614 model adopted in our design, according to its datasheet, demonstrates strong robustness against noise, voltage fluctuations, and device aging.

**Figure 5:**
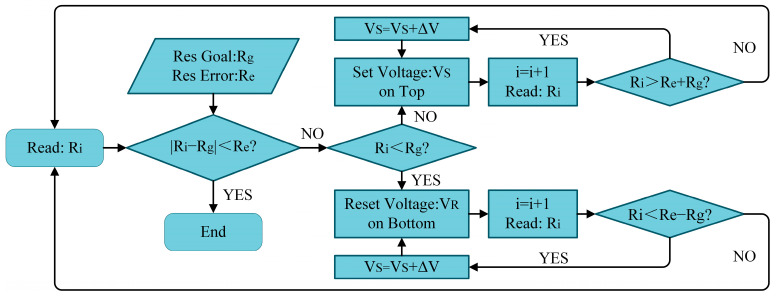
Flow chart of programming RRAM devices.

Another significant advantage of this read/write circuit is its ability to achieve precise resistance programming. The procedure for programming the memristor resistance is illustrated in Figure 5. First, a read operation is performed to measure the current resistance. The measured value is then compared with the target resistance within a predefined tolerance range to determine whether it meets the requirement. If not, the circuit decides whether a set or reset operation should be applied. Owing to the symmetric design of the circuit, the set process is described here as an example: a small set voltage is initially applied, followed by a read operation to check if the resistance meets the target. If the resistance is still outside the acceptable range, the set voltage is incrementally increased. This iterative process of applying set voltages and reading the resistance continues until the target value is achieved. If the memristor exhibits stable resistance states near the target value, this “write-read-verify” loop enables highly accurate resistance programming.

### 2.4. TiN/TiO_x_/HfO_2_/TiN RRAM

**Figure 6:**
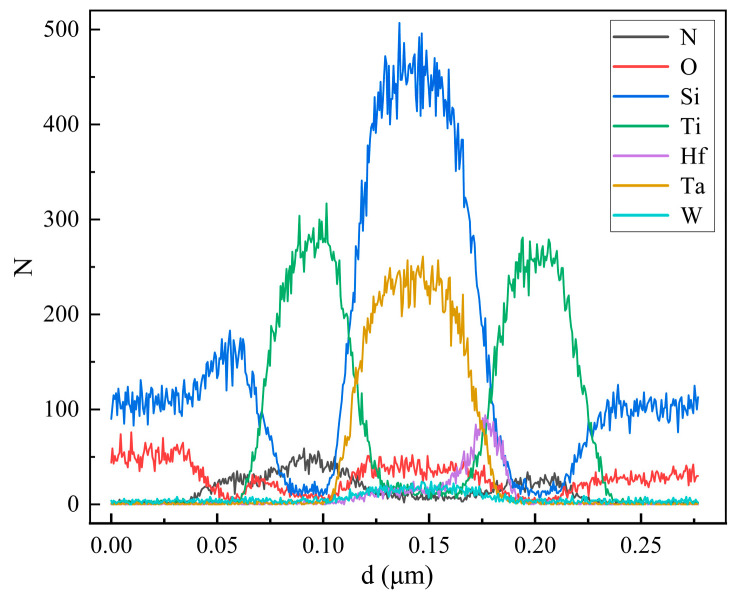
Memristor EDS line-scan profile.

This work is based on a 1T1R array architecture, which consists of 36 word lines for controlling transistor switching and 32 bit lines connected to the memristor cells. The source lines are interconnected to form a common-top-electrode structure. The transistors are fabricated using 180 nm process technology. Energy-Dispersive X-ray Spectroscopy (EDS) analysis was conducted on an individual memristor within the array, and the corresponding line-scan profile is presented in Figure 6. The results indicate that the memristor exhibits a multilayered TiN/TiOx/HfO_2_/TiN structure.

**Figure 7:**
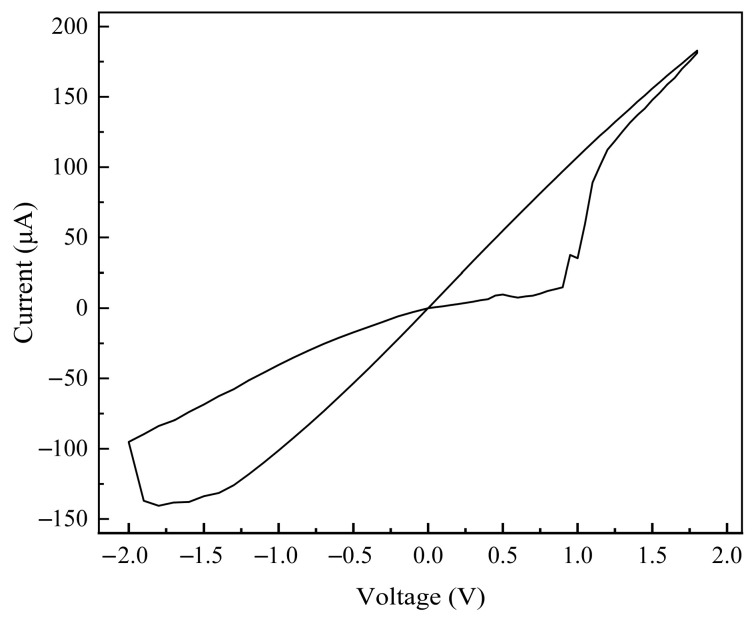
Hysteresis loop of the memristor.

The hysteretic current-voltage (I-V) curve of a selected memristor cell is shown in Figure 7. It can be observed that the device exhibits low operating voltages, with a set voltage of approximately 1 V and a reset voltage of about −1.5 V.

**Figure 8:**
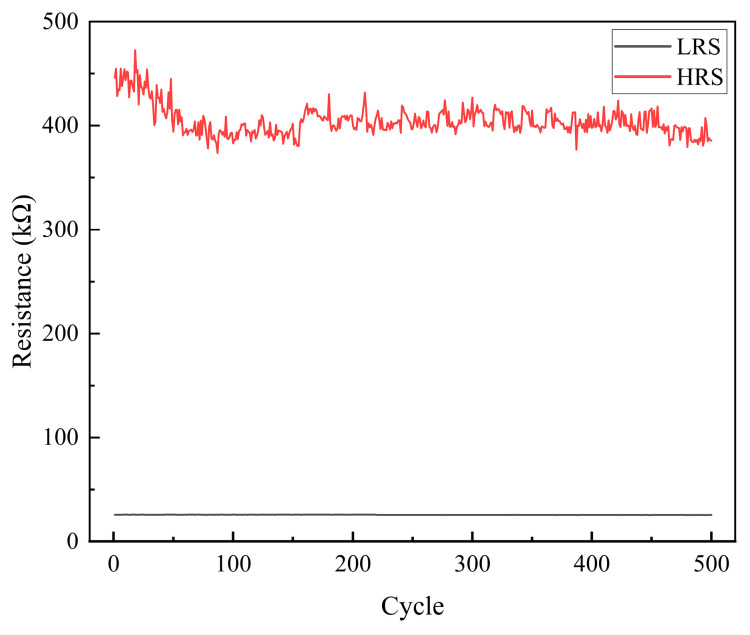
Cycling endurance characteristic of the memristor.

Figure 8 demonstrates that the memristor exhibits robust endurance, sustaining more than 500 stable write-erase cycles and indicating a reliable performance for repeated operations.

**Figure 9:**
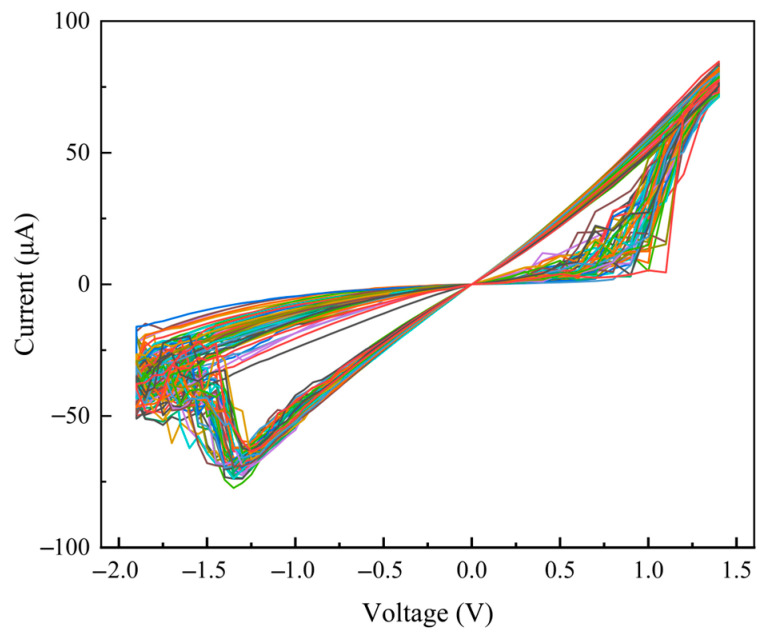
Device-to-Device consistency of the memristors.

In addition, Figure 9 displays the hysteresis curves of 50 memristors in the array, revealing good device-to-device consistency, which is key to achieving high computational accuracy in parallel operations.

## 3. Results

### 3.1. Memristor Resistance Distribution

As analyzed in Section 2.2, the memristor must exhibit at least eight stable and distinguishable conductance states to meet the requirements for 3-bit computing. Through a systematic study of 50 memristor devices and precise programming of their resistance states using the proposed read/write circuit, eight clearly distinguishable resistance levels were successfully identified.

**Figure 10:**
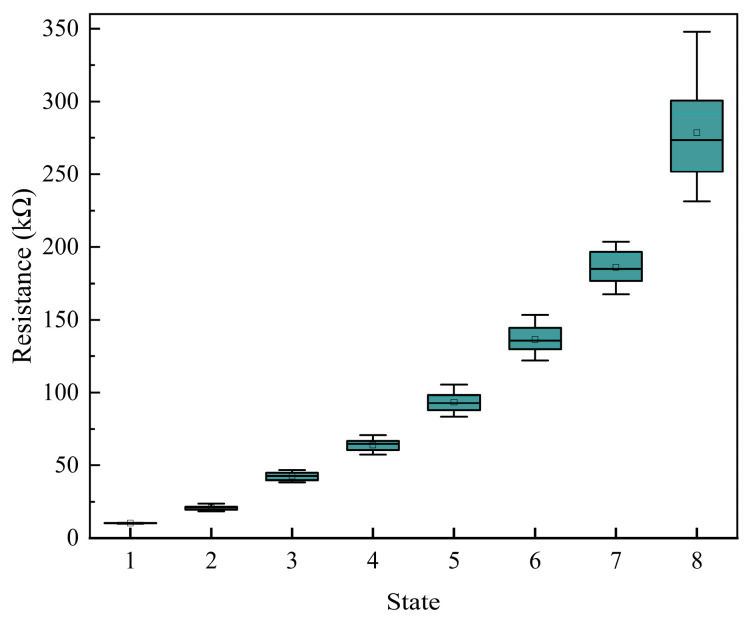
Box plot of memristor resistance distribution.

To illustrate the statistical results, the distributions of these eight resistance states across the 50 devices are presented in the form of box plots in Figure 10. Since the acceptable error margin for resistance programming was defined relative to the target resistance value, states with higher nominal resistances exhibit broader distributions.

For a quantitative summary, the corresponding statistical data (mean, standard deviation, and count) for each state are provided in Table 1. These results demonstrate satisfactory device-to-device uniformity for all eight resistance states.

**Figure 11:**
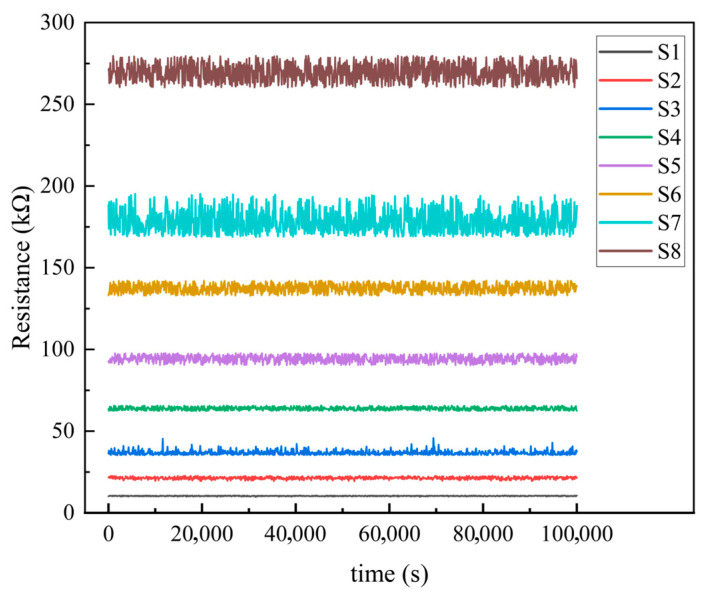
Retention characterization of the eight resistance states.

Furthermore, the retention characteristics of the eight resistance states are shown in Figure 11. The data exhibit minimal degradation over 10,000 s, with clear separation maintained between all states. This confirms that the states remain distinguishable after extended periods, which is critical for reliable system operation.

### 3.2. Voltage Waveforms

**Figure 12:**
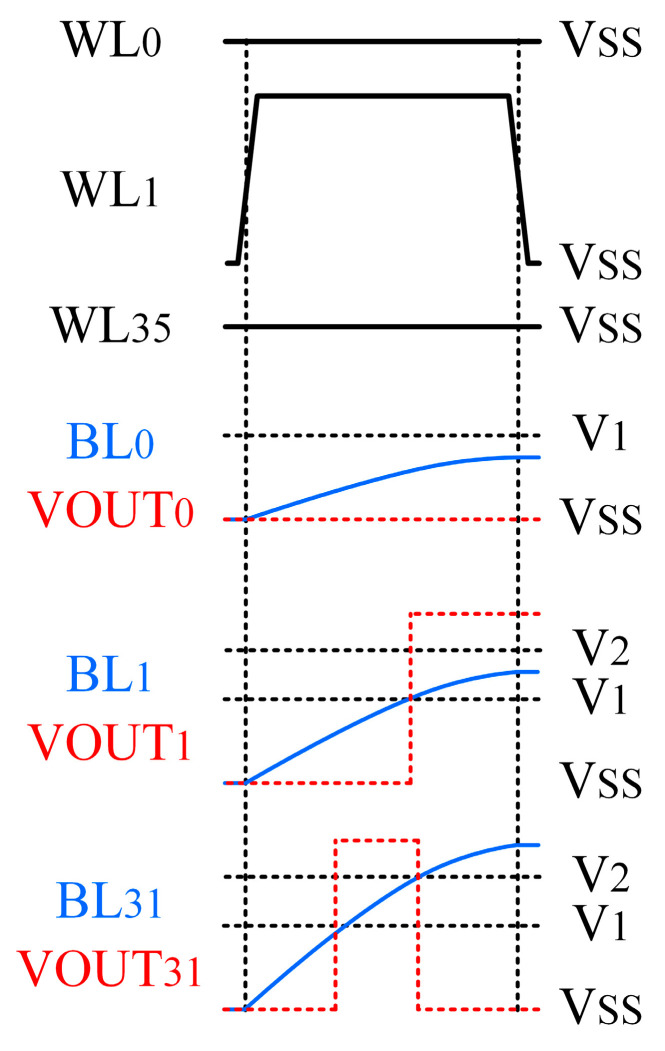
Bit Line and output voltage waveforms.

Identical capacitors are connected in series to each bit line, forming an RC signal path together with the memristors. Figure 12 shows the transient characteristics and output waveforms for memristors programmed to different resistance states. Since the resistance ranges corresponding to each state show no overlap, the readout circuit can effectively distinguish one or several target resistance states, provided that appropriate reference voltages are selected.

**Figure 13:**
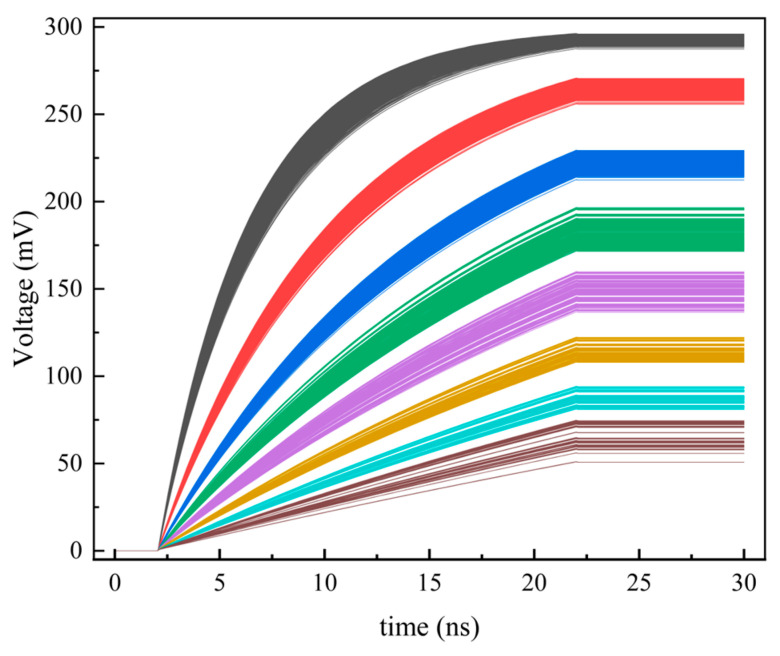
Waveform simulation for evaluating parasitic effects in a large-scale array.

Based on the memristor model, we evaluated the impact of parasitic effects and IR drops in large-scale arrays by performing Monte Carlo simulations. A read voltage of 0.3 V was applied to the memristor. The charging waveforms for the eight resistance states, which employed a 0.3 pF capacitor, are shown in Figure 13.

As shown in the results, the charging waveforms of the eight states in the large-scale array exhibit sufficient separation without overlap across various PVT conditions, with a minimum voltage difference of approximately 6.5 mV between the two closest states. In the 32 × 36 array demonstration, we employed the TLV3201 comparator, which has a typical input offset voltage of 1 mV. This ensures that the comparator can reliably distinguish between all eight resistance states.

Meanwhile, a 12-bit DAC (AD5326) was employed in the demonstration. With a 3.3 V reference voltage, it achieves a voltage resolution of 0.8 mV, which is sufficient to provide precise reference voltages for the readout circuit. The mapping between 3-bit input signals and reference voltages is exemplified in Table 2.

## 4. Discussion

### 4.1. Implementation of CAM/ACAM

Based on the above analysis, the proposed circuit can be applied to CAM systems. By setting the reference voltage according to the input signal, the circuit can precisely discriminate the target resistance state, while the target address is obtained through subsequent address encoding operations. In IoT edge devices, for example, massive amounts of data patterns can be pre-stored in the CAM. When sensor data matches one of these patterns, the device can immediately trigger a local alert or action without waking the main processor or transmitting data, significantly reducing both power consumption and latency [33,34].

Beyond exact matching against a specific resistance value, the circuit can also match a range of resistance states, making it applicable to ACAM. In many computing scenarios, locating entries that are sufficiently similar to the input often provides an adequate solution [35,36,37].

A representative application is in vector databases, where a core operation is the Approximate Nearest Neighbor Search (ANNS). In this scheme, database vectors are pre-loaded into the RRAM array. The components of the query vector are fed as input signals to the reference voltage generator, which subsequently produces the corresponding reference voltages applied to readout circuit. A matching operation is performed for each element by defining an acceptance window, [*V_1_*,*V_2_*], which acts as a similarity threshold. An element from a stored vector is considered a match if its corresponding output voltage falls within this range. This ternary matching behavior, characteristic of an ACAM, can be described as:

(3) $$M_{i,j}=\left\{\begin{array}{l} 1,\;\;\;\;\;\;\;\;\mathrm{if}\;V_{1}\;\leq \;V_{out,j}\;\leq \;V_{2}\\ 0,\;\;\;\;\;\;\;\;\mathrm{otherwise} \end{array}\right.$$

Here, *M_i,j_* represents the binary matching result of the *i*-th element on the *j*-th bit line, and *V_out,j_* is voltage of the *j*-th bit line.

**Figure 14:**
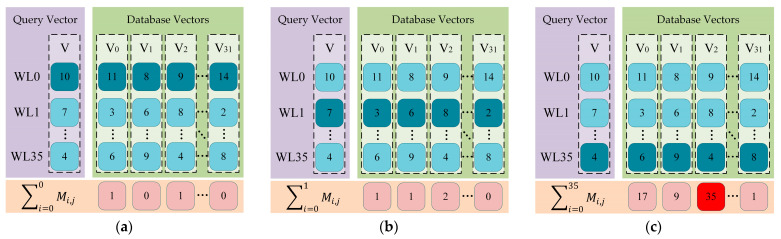
Schematic diagram of the nearest neighbor vector matching process. (**a**) Matching the first element of the query vector; (**b**) Matching the second element of the query vector; (**c**) Matching the 36-th element of the query vector and generating the total proximity score on each bit line.

Figure 14 shows a schematic diagram of the process for finding the nearest neighbor vectors. The total number of matched elements ($\sum_{i=0}^{35}M_{i,j}$) on the *j*-th bit line serves as a proximity score, indicating the similarity between the query vector and the stored vector. The vectors with the highest scores are identified as the nearest neighbors.

To enhance ACAM matching reliability, the acceptance window [*V_1,_ V_2_*] must be adaptively controlled to balance false positives against false negatives, thereby optimizing matching accuracy. We propose the following algorithmic strategies for dynamic threshold optimization:

1. Hierarchical Search Strategy: This approach employs a two-stage search pipeline to optimize for recall and precision, respectively. The first stage utilizes a larger voltage window to scan the entire memory array, aiming to generate a candidate set while minimizing missed detections. The second stage then applies a significantly narrower voltage window exclusively to this candidate set, performing a fine-grained search to select the optimal match by increasing the matching standard, thereby effectively reducing mismatches;
2. Feedback-based Closed-Loop Control: This strategy dynamically adjusts the voltage window in real-time based on system-monitored key metrics, such as the observed match rate or application-specific accuracy. The control algorithm will progressively tighten the threshold when a high mismatch rate is detected. Conversely, the threshold will be appropriately relaxed when a high missed-detection rate is suspected.

### 4.2. Performance Evaluation

This section conducts a systematic performance evaluation and analysis of the proposed architecture, specifically addressing the following aspects:

- Device Yield: In our previous study [17], the fundamental yield of devices was measured to be 99.5% (Figure 9 of the reference). In the current work, we conducted eight-state programming tests on 50 randomly selected devices, with results showing no overlap in the resistance distributions across all states for any device. Considering these findings collectively, although exhaustive testing of all devices was not performed, we can reasonably infer that the yield of devices capable of being reliably programmed into eight non-overlapping states is no less than 99%.
- Limits on Stable States: The number of distinguishable states in memristors is not unbounded, as it is constrained by several critical factors. First, the memristor′s resistance window ratio (the ratio between its high- and low-resistance states) directly determines the upper limit of achievable stable states. A larger window ratio theoretically enables more intermediate states to be distinguished. Second, due to device-to-device variations within an array, the specific resistance value of a target state is subject to fluctuations. Furthermore, the performance of peripheral circuits, such as the magnitude of the read voltage and the precision of the read/write circuit (including comparator resolution and reference voltage stability), collectively forms the ultimate bottleneck limiting the number of distinguishable states.
- Trade-offs in Matching Mode Switching: At the chip level, integrating multiple DACs would introduce significant power and area overhead. To mitigate this, the proposed design employs only a single reference voltage generator, which requires operational mode switching. If these constraints are disregarded, distinct reference voltages could be applied to different columns, enabling separate implementation of CAM and ACAM functions across columns and thereby avoiding the need for dynamic reference voltage adjustment. The primary limitation on mode switching speed lies in the settling time of the DAC within the reference voltage generator. According to the datasheet of the DAC used, this delay is on the order of 10 μs.
- Read/Write Circuit Performance: Experimental measurements demonstrate that the proposed read/write circuit achieves a minimum programming pulse width of 5 μs, representing a three-order-of-magnitude improvement in speed compared to the 5 ms minimum pulse width of the SMU in a Keithley 4200-SCS parameter analyzer. In terms of power consumption, the total power of the current board-level system measures in the milliwatt range. However, this metric is strongly influenced by Printed Circuit Board (PCB) parasitics and therefore does not faithfully represent the power efficiency of the core circuit in an integrated-circuit implementation. Nevertheless, the intrinsic power characteristics can be analyzed: During read operations, the memristor operates in a low-current steady state, resulting in low and stable power dissipation. For instance, with a read voltage of 0.2 V applied to the low resistance state of 10.19 kΩ, the current is merely 19.63 μA, yielding a power dissipation of only 3.93 μW; During write operations, since the amplitude of the applied voltage pulse is fixed, the instantaneous power consumption is primarily determined by the memristor’s conductance—increasing during Set and decreasing during Reset. Notably, except for possible current spikes during the initial forming process, no power surges occur in subsequent standard programming operations, ensuring system reliability.
- Assessment of the Digital Control Logic: The digital control logic comprises four core modules: a 3-bit PWM block, a 36-to-1 word-line decoder with two-stage logic, a 192-bit LUT built from register arrays (8 addresses × 24 bits), and a priority encoder for match processing and address generation. The total equivalent gate count of these components is estimated to be 540 logic gates. Compared to the core overhead of the analog array and readout circuit, the area proportion of this digital control logic is entirely within an acceptable range. It should be noted, however, that this is only a rough estimate, and the precise digital overhead requires further validation in subsequent IC implementation and fabrication. It is particularly important to emphasize that linearity deviations in the PWM module do not substantially impact the overall system performance. The core function of this module is to adaptively adjust the charging time based on fluctuations in the RC time constant to ensure computational accuracy. Even if its output exhibits certain non-linearity, this can be compensated for by appropriately expanding the tolerance range of the reference voltage, thereby guaranteeing the accuracy of the computational results. Consequently, the linearity of the PWM does not directly impact overall system performance. Furthermore, while the PWM control signal (generated by an Field-Programmable Gate Array, FPGA) exhibits fixed rise/fall times that systematically affect the effective pulse width, this fixed offset can be calibrated out. Additionally, with jitter at the nanosecond level—significantly smaller than the pulse widths used—its influence remains negligible.
- Performance Metrics Projection: This research primarily focuses on proposing a novel computing-in-memory paradigm and demonstrating its proof-of-concept validation. Given the current stage of development, a comprehensive system-level performance evaluation has not yet been conducted. However, based on existing experimental data, the following performance metrics can be prospectively estimated:
  1. Latency: Based on the circuit architecture presented, the primary sources of delay reside in the RC charging phase and comparator response. With reference to the approximately 20 ns charging delay shown in Figure 13 and the typical 40 ns delay specified in the comparator datasheet, we estimate the system′s single-operation latency to be within 100 ns;
  2. Power Consumption: Due to the inclusion of non-core circuit power consumption such as PCB parasitics in the current board-level implementation, the measured total energy efficiency cannot accurately represent the level achievable in an integrated circuit implementation. Based on the principles of computing-in-memory and recent related research, we estimate that the energy consumption per search operation of this scheme could reach the tens of picojoules level when implemented as an integrated circuit.
  3. Error Rates: System level bit error rate statistics under continuous real data streams have not been conducted in this study. However, key parameters obtained from current testing, including device stability and consistency as well as the precise writing procedures of the read/write circuit, form the fundamental basis for ensuring computational accuracy.
  4. Throughput Consideration: The present proof-of-concept demonstration, implemented on a small-scale memristor array, does not support comprehensive evaluation of system throughput. Accurate characterization of throughput performance will require extensive system-level simulations of large-scale arrays in future work, which represents a critical direction for our subsequent research.

### 4.3. Scalability Challenges and Future Perspectives

As memristor-based computing-in-memory architectures advance toward large-scale arrays, a series of technical challenges emerges:

- Array Scalability Constraints: With increasing array row count, the parallel parasitic capacitance along bit lines raises the total capacitive load, directly affecting the RC time constant and introducing systematic deviations in multi-level computation accuracy. Concurrently, as the array column count expands, the growing load capacitance at the reference voltage node degrades the DAC′s settling speed, consequently constraining the dynamic response of CAM/ACAM mode switching.
- Multi-Level Storage Stability: While the current read/write circuit meets the resistance programming requirements in the 50-device test array, significant challenges emerge with array scaling or increased state numbers. Device-to-device variations become more pronounced under these conditions, imposing stricter demands on programming accuracy and tolerance control during write-verify operations. Furthermore, read disturbance effects introduce additional resistance drift, particularly affecting low-resistance states which demonstrate higher sensitivity to electrical stress. The inherent device disparities cause broadening of resistance distributions, directly compromising the reliability and state distinguishability in multi-level storage.
- Coding Time Overhead: As the scale increases, the most significant growth occurs in device programming time. Performing write-verify operations sequentially for each device in the array would require prohibitively long durations, severely constraining coding efficiency.
- Process Integration: First, the heterogeneous integration of memristors with standard CMOS processes inherently faces compatibility issues in materials and thermal budgets. Second, as the array scale increases, minor process variations become significantly amplified, causing substantial deviations in device characteristics across different regions and severely compromising overall uniformity. Finally, during packaging, the thermal effects of packaging materials on the temperature distribution of the array further exacerbate performance variations among devices, making system-level performance optimization exceedingly complex.

To advance the proposed system, we outline the following optimization pathways across multiple levels:

1. Device-Level Enhancements: We will explore memristor materials with wider conductance windows and a greater number of distinguishable states, while conducting in-depth research to improve device-to-device uniformity within arrays. A systematic investigation into device reliability will be performed to understand failure statistics and mechanisms, enabling circuit designs that inherently compensate for these effects. Furthermore, we will develop memristor material systems with improved compatibility with standard CMOS processes to facilitate large-scale integration;
2. Circuit-Level Innovations: The read/write circuit will be enhanced to support memristors with increased state capacity, achieving sub-millivolt precision in read/write operations. These improvements will be coupled with adaptive compensation algorithms to suppress system-level variations. Additionally, we will develop novel write circuits capable of programming multiple memristors in parallel, significantly improving programming efficiency for large-scale arrays.
3. Algorithm and Architecture Improvements: We will refine the approximate matching algorithms for ACAM to enhance robustness against noise and drift-induced false positives/negatives. To address reliability concerns from device failures, we will incorporate system-level protection mechanisms such as Error-Correcting Codes (ECC) and redundancy schemes.
4. Implementation and Validation: Our ultimate goal is to realize the complete system architecture through Application-Specific Integrated Circuit (ASIC) implementation to validate its efficacy in practical applications. In specific biosensing scenarios such as wearable sweat monitoring [38] and single-cell electrochemotherapy efficacy assessment [39], our computing-in-memory architecture can serve as the core data processing unit. By integrating with such highly sensitive biosensor front-ends, a complete sensing-computation-decision system can be constructed: raw physiological data captured by the sensors (e.g., resonant frequency shifts caused by sweat composition changes, or variations in cellular dielectric properties) can be directly processed through real-time, high-efficiency matching and recognition in our ACAM system, enabling immediate assessment of physiological status or treatment effectiveness on edge devices. This implementation pathway will be co-optimized with ultra-low-power design to meet the stringent energy efficiency requirements of such long-term, autonomous monitoring applications [40]. We plan to conduct comprehensive characterization and validation of system performance (including latency, throughput, energy efficiency, error rate, and area) under these biomedical monitoring paradigms post-tape-out.

## 5. Conclusions

This paper presents a reconfigurable RRAM CIM architecture capable of implementing multi-bit content-addressable memory. The design fully leverages the analog multi-level resistance characteristics of memristors, overcoming the limitations of traditional binary computing and significantly improving storage density and computational parallelism. To achieve accurate programming of memristor conductance states, a portable read/write circuit is introduced alongside a closed-loop write-verify strategy, which substantially improves the reliability and precision of multi-bit operations. Moreover, by incorporating a configurable LUT to dynamically adjust reference voltages, the circuit can flexibly switch between exact-matching CAM and approximate-matching ACAM suitable for similarity searches, greatly expanding its application scenarios in edge computing.

Experimental results demonstrate that the 32 × 36 memristor array based on TiN/TiOx/HfO_2_/TiN structure exhibits eight stable and distinguishable resistance states, with excellent retention characteristics and device-to-device consistency. Large-scale array simulations further confirm that, under various process, voltage, and temperature conditions, the minimum voltage spacing between charging waveforms of different states exceeds 6.5 mV, ensuring reliable discrimination by the readout circuit.

In summary, the proposed scheme provides a highly promising hardware prototype for intelligent edge computing in next-generation sensor networks. Future research will focus on addressing scalability challenges associated with large-scale arrays and conducting comprehensive system-level validation through ASIC implementation in specific biosensing application scenarios.

## Figures and Tables

**Table 1:** Statistical parameters of the multi-level resistance states.

State	Device Count	Mean (kΩ)	Standard Deviation (kΩ)
State 1	50	10.19	0.17
State 2	50	20.80	1.38
State 3	50	42.49	2.81
State 4	50	63.98	3.83
State 5	50	93.39	6.64
State 6	50	136.57	9.14
State 7	50	186.08	11.12
State 8	50	278.55	32.47

**Table 2:** Mapping between 3-bit input signals and reference voltages.

State	3-bit Input Signals	Reference Voltages [V_1_, V_2_] (mV)
State 1	000	[0, 78]
State 2	001	[78, 101]
State 3	010	[101, 130]
State 4	011	[130, 166]
State 5	100	[166, 206]
State 6	101	[206, 244]
State 7	110	[244, 280]
State 8	111	[280, 300]

## Data Availability

The data presented in this study are available on request from the corresponding author.

## Abbreviations

The following abbreviations are used in this manuscript:

ACAM	Approximate Content-Addressable Memory
ADC	Analog-to-Digital Converter
ANNS	Approximate Nearest Neighbor Search
ASIC	Application-Specific Integrated Circuit
CAM	Content-Addressable Memory
CIM	Computing-In-Memory
CMOS	Complementary Metal-Oxide-Semiconductor
DAC	Digital-to-Analog Converter
DUT	Device Under Test
ECC	Error-Correcting Codes
EDS	Energy Dispersive X-ray Spectroscopy
FPGA	Field-Programmable Gate Array
IoT	Internet of Things
LLMs	Large Language Models
LUT	Look-Up Table
MCU	Microcontroller Unit
MUX	Multiplexer
PCB	Printed Circuit Board
PWM	Pulse-Width Modulation
RRAM	Resistive Random-Access Memory
1T1R	One-Transistor-One-RRAM

## References

[1] Zhang J., Sadredini E. Inhale: Enabling High-Performance and Energy-Efficient In-SRAM Cryptographic Hash for IoT. Proceedings of the 2022 IEEE/ACM International Conference on Computer Aided Design (ICCAD).

[2] Lu X., Chen S., Pang Z., Sun S., Yin X., Zhuo C. In-Memory Multi-Bit Multiplication and Accumulation (MAC) Using FeFET for Energy Efficient IoT. Proceedings of the 2022 2nd International Conference on Intelligent Technology and Embedded Systems (ICITES).

[3] Li H., Shou G. Synergetic Node of Edge Computing and Hybrid Fibre-Wireless (FiWi) Access Networks for IoT. Proceedings of the 2019 IEEE 30th Annual International Symposium on Personal, Indoor and Mobile Radio Communications (PIMRC).

[4] Shuvo M.M.H., Islam S.K., Cheng J., Morshed B.I. (2022). Efficient Acceleration of Deep Learning Inference on Resource-Constrained Edge Devices: A Review. Proc. IEEE.

[5] Marchesin A., Turvani G., Coluccio A., Riente F., Vacca M., Roch M.R., Graziano M., Zamboni M. Octantis: An Exploration Tool for Beyond von Neumann architectures. Proceedings of the 2021 16th International Conference on Design & Technology of Integrated Systems in Nanoscale Era (DTIS).

[6] Marchesin A., Naclerio A., Riente F., Graziano M. (2024). Beyond Von Neumann Architectures: Exploring Algorithmic Opportunities via Octantis. IEEE Access.

[7] Hsu H.-H., Wen T.-H., Wu P.-C., Jhang C.-J., You D.-Q., Chen P.-C., Chang M.-F. Challenges in Circuits of Nonvolatile Compute-In-Memory for Edge AI Chips. Proceedings of the 2023 IEEE 66th International Midwest Symposium on Circuits and Systems (MWSCAS).

[8] Xue C.-X., Chang M.-F. Challenges in Circuit Designs of Nonvolatile-memory based computing-in-memory for AI Edge Devices. Proceedings of the 2019 International SoC Design Conference (ISOCC).

[9] Si X., Xue C.-X., Su J.-W., Zhang Z., Li S.-H., Sheu S.-S., Lee H.-Y., Chen P.-C., Wu H., Qian H. Circuit Design Challenges in Computing-in-Memory for AI Edge Devices. Proceedings of the 2019 IEEE 13th International Conference on ASIC (ASICON).

[10] Chang L., Li C., Zhao X., Zhu Z., Tong Y., Lin S., Zhou J. Trend of Emerging Non-Volatile Memory for AI Processor. Proceedings of the 2021 18th International SoC Design Conference (ISOCC).

[11] Shooshtari M., Kim S., Pahlavan S., Rivera-Sierra G., Través M.J., Serrano-Gotarredona T., Bisquert J., Linares-Barranco B. (2025). Advancing Logic Circuits with Halide Perovskite Memristors for Next-Generation Digital Systems. SmartMat.

[12] Wang Z., Nalla P.S., Krishnan G., Joshi R.V., Cady N.C., Fan D., Seo J.-S., Cao Y. Digital-Assisted Analog In-Memory Computing with RRAM Devices. Proceedings of the 2023 International VLSI Symposium on Technology, Systems and Applications (VLSI-TSA/VLSI-DAT).

[13] Gu H., Wang Z., Li J., Ding H., Shan L., Bao L., Liang L., Cai Y., Huang R. Invited Paper: Design of an RRAM In-Memory Computing Scheme for Target Tracking Applications. Proceedings of the 2024 IEEE International Conference on Integrated Circuits, Technologies and Applications (ICTA).

[14] Deng J., Zhou K., Yang H., Yu C., Yang J. A High-Density RRAM-Based Ising Machine with Analog In-Memory Operation for Solving Combinatorial Optimization Problems. Proceedings of the 2025 IEEE International Symposium on Circuits and Systems (ISCAS).

[15] Guo H., Li Y., Ren T., Dong C., Wang L., Zhao Y., Zhang Y. A RRAM Based 9T1R NVSRAM for Low-Power Computing in Memory. Proceedings of the 2024 IEEE 17th International Conference on Solid-State & Integrated Circuit Technology (ICSICT).

[16] Dongre A., Boro B., Trivedi G. (2023). ADC-Less Reprogrammable RRAM Array Architecture for In-Memory Computing. IEEE Trans. Very Large Scale Integr. (VLSI) Syst..

[17] Wang J., Zhang T., Liu S., Liu Y., Wu Y., Hu S., Chen T., Liu Y., Yang Y., Huang R. (2023). Design and Implementation of a Hybrid, ADC/DAC-Free, Input-Sparsity-Aware, Precision Reconfigurable RRAM Processing-in-Memory Chip. IEEE J. Solid-State Circuits.

[18] Moorthii C., Singla A., Suri M. DNA-CIM: DNA Sequence Analysis Using RRAM-Based Compute In-Memory Accelerator. Proceedings of the 2025 38th International Conference on VLSI Design and 2024 23rd International Conference on Embedded Systems (VLSID).

[19] Oh H., Kim H., Kang N., Kim Y., Park J., Kim J.-J. Single RRAM Cell-based In-Memory Accelerator Architecture for Binary Neural Networks. Proceedings of the 2021 IEEE 3rd International Conference on Artificial Intelligence Circuits and Systems (AICAS).

[20] Jia R., Pechmann S., Markus F., Wenger C., Zhang L., Hagelauer A. Soft-Error Analysis of RRAM 1T1R Compute-In-Memory Core for Artificial Neural Networks. Proceedings of the 2024 39th Conference on Design of Circuits and Integrated Systems (DCIS).

[21] Wang Z., Li Y., Su Y.-T., Zhou Y., Yin K., Cheng L., Chang T.-C., Xue K., Sze S., Miao X. Implementation of Functionally Complete Boolean Logic and 8-Bit Adder in CMOS Compatible 1T1R RRAMs for In-Memory Computing. Proceedings of the 2018 IEEE International Memory Workshop (IMW).

[22] Prakash A., Park J., Song J., Woo J., Cha E.-J., Hwang H. (2014). Demonstration of Low Power 3-bit Multilevel Cell Characteristics in a TaOx-Based RRAM by Stack Engineering. IEEE Electron Device Lett..

[23] Zhang F., Zhang H., Shrestha P., Zhu Y., Maize K., Krylyuk S., Shakouri A., Campbell J., Cheung K., Bendersky L. An Ultra-fast Multi-level MoTe2-based RRAM. Proceedings of the 2018 IEEE International Electron Devices Meeting (IEDM).

[24] Yu D., Liu L.F., Chen B., Zhang F.F., Gao B., Fu Y.H., Liu X.Y., Kang J.F., Zhang X. Multilevel resistive switching characteristics in Ag/SiO2/Pt RRAM devices. Proceedings of the 2011 IEEE International Conference of Electron Devices and Solid-State Circuits.

[25] Liu Y.H., Wang J.J., Wang H.Z., Liu S., Wu Y.C., Hu S.G., Yu Q., Liu Z., Chen T.P., Yin Y. (2023). Braille recognition by E-skin system based on binary memristive neural network. Sci. Rep..

[26] He W., Yin S., Kim Y., Sun X., Kim J.-J., Yu S., Seo J.-S. (2020). 2-Bit-Per-Cell RRAM-Based In-Memory Computing for Area-/Energy-Efficient Deep Learning. IEEE Solid-State Circuits Lett..

[27] He W., Shim W., Yin S., Sun X., Fan D., Yu S., Seo J.-S. Characterization and Mitigation of Relaxation Effects on Multi-level RRAM based In-Memory Computing. Proceedings of the 2021 IEEE International Reliability Physics Symposium (IRPS).

[28] Li J., Wang Z., Wang C., Cai Y., Huang R. Design Considerations of Multi-Level 1S1R Cell for In-Memory Computing. Proceedings of the 2023 IEEE International Conference on Integrated Circuits, Technologies and Applications (ICTA).

[29] Esmanhotto E., Hirtzlin T., Castellani N., Martin S., Giraud B., Andrieu F., Nodin J.F., Querlioz D., Portal J.-M., Vianello E. Experimental demonstration of Single-Level and Multi-Level-Cell RRAM-based In-Memory Computing with up to 16 parallel operations. Proceedings of the 2022 IEEE International Reliability Physics Symposium (IRPS).

[30] Zhang Q., Yan L., Tao Y., Huang R., Yang Y. On-Chip Write & Verify and Endurance Enhancer Circuits towards Multi-level RRAM Array. Proceedings of the 2024 8th IEEE Electron Devices Technology & Manufacturing Conference (EDTM).

[31] Sun J., Wang Z., Gao J., Shan L., Wang Q., Yang Y., Cai Y., Huang R. ASAP: An Efficient and Reliable Programming Algorithm for Multi-level RRAM Cell. Proceedings of the 2024 IEEE International Reliability Physics Symposium (IRPS).

[32] Pechmann S., Hagelauer A. A Read Circuit Design for Multi-Level RRAM Cells Exhibiting Small Resistance Windows. Proceedings of the 2022 IEEE 65th International Midwest Symposium on Circuits and Systems (MWSCAS).

[33] Yang K. (2020). A Dual-Port 8-T CAM-Based Network Intrusion Detection Engine for IoT. IEEE Solid-State Circuits Lett..

[34] Almazrouei O., Magalingam P., Hasan M.K., Almehrzi M., Alshamsi A. Penetration Testing for IoT Security: The Case Study of a Wireless IP Security CAM. Proceedings of the 2023 IEEE 2nd International Conference on AI in Cybersecurity (ICAIC).

[35] Garzón E., Yavits L., Teman A., Lanuzza M. (2023). Approximate Content-Addressable Memories: A Review. Chips.

[36] Shi J., Qian W. Implementing Boolean Function by Ternary Content Addressable Memory with Approximate Match. Proceedings of the 2023 China Semiconductor Technology International Conference (CSTIC).

[37] Garzón E., Golman R., Lanuzza M., Teman A., Yavits L. (2023). A Low-Complexity Sensing Scheme for Approximate Matching Content-Addressable Memory. IEEE Trans. Circuits Syst. II Express Briefs.

[38] Wang S., Shen Y., Zheng Y. (2025). Coplanar Hybridization of Half-Mode Magnetic Plasmonic Skyrmions: Realization and Application in a Flexible Wearable Microwave Sweat Metasensor. IEEE Trans. Microw. Theory Tech..

[39] Tamra A., Zedek A., Rols M.-P., Dubuc D., Grenier K. (2022). Single Cell Microwave Biosensor for Monitoring Cellular Response to Electrochemotherapy. EEE Trans. Biomed. Eng..

[40] Citroni R., Mangini F., Frezza F. (2024). Efficient Integration of Ultra-low Power Techniques and Energy Harvesting in Self-Sufficient Devices: A Comprehensive Overview of Current Progress and Future Directions. Sensors.

